# Discoveries: an innovative platform for publishing cutting-edge research discoveries in medicine, biology and chemistry

**DOI:** 10.15190/d.2013.1

**Published:** 2013-12-31

**Authors:** Octavian Bucur, Alexandru Almasan, Barbara S. Nikolajczyk, Garth L. Nicolson, Jack Lawler, Victor E. Velculescu, Sorin Draghici, Mircea Leabu, Dorina Avram, Ilie Bucur, Enzo Calautti, George A. Calin, Subhash C. Chauhan, Mihai Ciubotaru, Stefan N. Constantinescu, Dipak Datta, Dan G. Duda, Mark T. Friedman, Paul J. Galardy, Brent T. Harris, Maite Huarte, Ahmad M. Khalil, Dario Marchetti, Liviu Movileanu, Roxana Nat, Carmelo Nucera, Aurel Popa-Wagner, Andreea L. Stancu, Shudong Zhu, Elisa A. Liehn

**Affiliations:** Department of Pathology, Harvard Medical School and Beth Israel Deaconess Medical Center, Boston, MA, USA; Department of Cancer Biology, Lerner Research Institute & Department of Radiation Oncology, Taussig Cancer Institute, Cleveland Clinic, Cleveland, Ohio, United States of America; Boston University School of Medicine, Department of Microbiology, Boston, MA, USA; The Institute for Molecular Medicine, Department of Molecular Pathology, Huntington Beach, CA, USA; Division of Cancer Biology and Angiogenesis, Department of Pathology, Beth Israel Deaconess Medical Center and Harvard Medical School, Boston, MA, USA; Sidney Kimmel Comprehensive Cancer Center, Johns Hopkins University, Baltimore, MD 21287; Intelligent Systems and Bioinformatics Laboratory, Wayne State University, Detroit, MI, USA; Department of Cellular and Molecular Medicine, University of Medicine and Pharmacy "Carol Davila" and "Victor Babes" National Institute of Pathology, Bucharest, Romania; Center for Cell Biology and Cancer Research & Center for Immunology and Microbial Disease, Albany Medical College, Albany, NY, USA; Applied Systems, Craiova, Romania; Department of Molecular Biotechnology and Health Sciences, University of Turin, Turin, Italy; Department of Experimental Therapeutics and Leukemia & Center for RNA Interference and Non-Coding RNAs, University of Texas MD Anderson Cancer Center, Houston, TX, USA; Department of Pharmaceutical Sciences & Center for Cancer Research, University of Tennessee Health Science Center, Memphis, Tennessee; Department of Immunobiology, Yale University School of Medicine, New Haven, CT, USA; de Duve Institute, Université Catholique de Louvain, Brussels, Belgium; Ludwig Institute for Cancer Research, Brussels, Belgium; CSIR-Central Drug Research Institute, Biochemistry Division, Lucknow, UP, India; Steele Laboratory for Tumor Biology, Department of Radiation Oncology, Massachusetts General Hospital & Harvard Medical School, Boston, MA, USA; Department of Pathology, St. Luke's-Roosevelt Hospital Center, Beth Israel Medical Center and Icahn School of Medicine at Mount Sinai, New York, NY, USA; Department of Biochemistry and Molecular Biology & Division of Pediatric Hematology/Oncology, Mayo Clinic, Rochester, MN, USA; Departments of Pathology and Neurology, Georgetown University Medical Center, Washington, DC, USA; Center for Applied Medical Research, University of Navarra, Pamplona, Spain; Department of Genetics, Center for RNA Molecular Biology, Case Western Reserve University School of Medicine, Cleveland, OH, USA; Departments of Molecular & Cellular Biology, Baylor College of Medicine, Houston, Texas, United States of America; Department of Physics, Structural Biology, Biochemistry, and Biophysics Program & Syracuse Biomaterials Institute, Syracuse University, Syracuse, NY, USA; Institute for Neuroscience, Medical University Innsbruck, Innsbruck, Austria; Human Thyroid Cancers Preclinical and Translational Research Laboratory, Experimental Division of Cancer Biology and Angiogenesis, Department of Pathology, Beth Israel Deaconess Medical Center and Harvard Medical School, Boston, MA, USA; Department of Psychiatry, Rostock University Medical School, Rostock, Germany; School of Biological Science and Technology, State Key Laboratory of Medical Genetics, Central South University, Changsha, China; Institute for Molecular Cardiovascular Research (IMCAR), RWTH Aachen University, Germany

**Keywords:** breakthrough research, interdisciplinary, multidisciplinary, integrative, journal, Molecular and Cellular Biology, Biochemistry, Biophysics, Genomics, Proteomics Biotechnology, Synthetic Biology, Systems Biology, Bioinformatics, Translational Medicine, Clinical findings, Clinical trials, Epidemiology, Global Medicine, Organic Chemistry, Inorganic Chemistry, Physical Chemistry, Ethics in Science

## Abstract

Discoveries is a new peer-reviewed, open access, online multidisciplinary and integrative journal publishing high impact reviews, experimental articles, perspective articles, and editorials from all areas related to medicine, biology, and chemistry, including but not limited to: Molecular and Cellular Biology, Biochemistry, Biophysics, Genomics, Proteomics, Biotechnology, Synthetic Biology, Bioengineering, Systems Biology, Bioinformatics, Translational Medicine, Medicine/ Clinical findings, Cognitive Science, Epidemiology, Global Medicine, Family Medicine, Organic/ Inorganic/ Physical Chemistry and Ethics in Science. Discoveries brings to the research community an outstanding editorial board that aims to address several of the innovations proposed above: there is no need to format the manuscript before submission, we have a rapid and efficient submission process, there is no need for a Cover Letter and we support the need for rules for validation of critical reagents, such as antibodies. Discoveries will aim to support high quality research on human subjects materials to provide relevance for non-human studies along with mechanistic insights into human biology and chemistry. We also aim to avoid requesting unnecessary experiments during the review process, without affecting the quality and conclusions of published manuscripts. In addition, we recognize the need of adopting the recommendations made by NCCD and other similar scientific guiding entities.

## Why do we need a new multidisciplinary publishing platform?

Over the last decades, we have witnessed an extraordinary explosion of scientific breakthroughs that have been successfully applied in many areas of research. In particular, rapid developments in fields such as medicine, biology, chemistry and related disciplines provides an unprecedented opportunity for scientists to be involved in this scientific revolution and enthusiastically contribute to it with life-changing discoveries. This accelerated evolution has led to an exponentially increased number of published research articles. Although there has been a surprisingly high number of newly launched journals in the last several years (trying to keep up with the increasing number of submitted research manuscripts), only a small fraction of these journals are ISI/PubMed indexed and have meaningful impact factors. Only few journals can be considered as high impact factor and very few of the recently launched journals have reached this stage. Moreover, there is an even smaller number of new high quality multidisciplinary journals covering more than one area of research. An increasing need for interdisciplinary approaches makes publication of high quality, innovative and reproducible papers in non-multidisciplinary journals sometimes challenging.

With a dismal acceptance rate of less than 8% in some of the top multidisciplinary journals, such as Science, Nature, other journals from the Nature family, the New England Journal of Medicine^1-3^ many of the cutting-edge discoveries not accepted for publication end up being re-submitted (frequently multiple times) to a lower impact factor journal, most of the time a more specialized one. In addition, a low acceptance rate also makes the reviewing process prone to subjectivity and may result in outstanding manuscripts being rejected. Thus, we consider that new high impact platforms for publishing high-quality interdisciplinary/ multidisciplinary research and cutting-edge discoveries are very much needed.

## Why is the submission process so cumbersome? The need for innovation:

### 1. Why does a manuscript have a specific format before it is even accepted?

Many manuscripts submitted in a specific format requested by a particular journal are eventually rejected. For a journal with an acceptance rate of less than 8%, the vast majority of the manuscripts will subsequently be submitted to an alternative journal, with different formatting requirements. In fact, many manuscripts are submitted to several journals before they are finally accepted. Thus, the authors spend *considerable* and *unnecessary* time modifying the format of the manuscript every time when they submit it to a different journal. We believe that this cumbersome and multi-step submission process increases the submission time more than is necessary. **

*Would it not be more efficient for journals to consider reviewing manuscripts in different formats and only request the specific format for those accepted, after completion of the reviewing process and a final decision was reached?*

*Why make more than 92% of the authors with rejected manuscripts (submitted to top journals) spend unnecessary time to format them, only to end up changing the format again before the manuscript is published?*

Our solution is simple and straightforward: allow submission of manuscripts in different formats and only request the re-formatting for those accepted, after reviewing process is finalized.

### 2. Why not utilize a rapid and simple submission process?

We believe that an easy and simplified submission process will be well received by authors.

** *Why not allow authors to submit their manuscripts with only minimal necessary information in less than a few minutes? In addition, why not complete the additional agreements or other information that is requested after acceptance? What is the point of submitting a Cover Letter with a letterhead and signature by the corresponding author when that author can just conveniently write the same text and send it in an e-mail (with or without an electronic signature) or can submit it as a text on the submission platform?*

### 3. Is a Cover Letter really necessary?**

*Do we really need a Cover Letter, when we have the Abstract and Conclusions of a manuscript in front of us?* Many editors and reviewers consider a Cover Letter useful, since it is easier for them to get a quick general idea about the scope and importance of the manuscript. Authors may consider that the Cover Letter is a way of highlighting their discoveries and significance of their findings. However, in our opinion, it is faster, easier and without significant consequences for authors to submit a manuscript without a Cover Letter, as long as the innovation, novelty, and importance of their findings is well underscored in the Abstract and Conclusion sections of the manuscript. Thus, we think that the Cover Letter should be optional.

### 4. Are rules for validation of key antibodies, siRNAs, and other reagents employed essential?

Antibodies, other reagents, inadequate animal models or cell-based models/assays, insufficient number of experimental repeats and statistical inadequacies all contribute to the low reproducibility of preclinical and clinical data^4-8^. Thus, adequate measures and rules for reagents defined by the journals would be of great help in decreasing the number of reported irreproducible research findings.

A recent study pointed out that many of the antibodies used in publications are non-specific and some of them do not even detect endogenous target proteins in multiple cell lines^4^. The authors should employ knockdown or knockout validation experiments to confirm the specificity of the employed antibodies. Thus, requesting proofs of the specificity of the main/key antibodies used in the manuscript would be of great help. These proofs may be the experiments performed by the authors themselves, results already published or validation experiments done by the manufacturer. Also, it is worth mentioning a recent initiative for Independent Antibody Validation to Improve Research Quality^9^. Manufacturers and researchers can send antibodies to be validated by an independent laboratory.

Due to the numerous off-target effects of siRNA or shRNAs widely used in preclinical biomedical research, rescue experiments, or at least two different types of siRNA/shRNAs for the same target, need to be used. Many journals, reviewers or editors still accept manuscripts that employ only one siRNA/shRNA.

These are only two important examples. Several journals, such as those published by American Association for Cancer Research, already have rules for validation of antibodies and other reagents^10^.

Therefore, the articles submitted to Discoveries are expected to describe work undertaken only with critical reagents that have been validated using one of the manners suggested above, or accompanied by other convincing scientific evidence about their effects.

### 5. Is there a need for new scientific research rules and for joining existing initiatives?

We want to highlight the importance of using standards in scientific research, such as the definition of cell death and classificasion of all cell death types^11-13^. Notable examples are the recommendations on classification of cell death made by Nomenclature Committee on Cell Death (NCCD)^11,12^. One of the committee’s suggestions is to use at least two different methods for detection of apoptosis or other forms of cell death^11^, since it is well established that many of the methods that detect apoptosis may also detect necrosis^13^. A second recommendation is to avoid terminology such as “% apoptosis”, “% cell death”, or “% survival”. Instead, the authors should mention exactly what they have measured by stating the employed method, such as “% cells TUNEL positive”, or “% cells Annexin V positive” ^11,13^. This is due to the fact that most of the techniques are not entirely specific for detection of apoptosis, cell death, survival, or proliferation. For example, MTT/MTS assays are considered methods that can measure survival, proliferation, or a combination of survival and proliferation, while Annexin V staining alone can detect not only apoptosis, but also non-apoptotic cell death, such as necrosis^13^. NCCD “urges” all scientific journals to join NCCD and Cell Death and Differentiation journal’s initiative in adopting these recommendations^11,13^. Similar initiatives and recommendations are needed in many other areas of research.

### 6. Can we avoid requesting unnecessary experiments during the reviewing process?

*How many of the experiments suggested by the reviewers/editor are indeed necessary to accept a manuscript?* The answer to this question depends on many variables, including the standards of the journal and the quality of the experiments presented in the manuscript. The myth of the complete paper enlists effort from entire laboratories so that a complete paper is eventually published, rather than one experimental observation that would be sufficient in itself to advance the field and benefit others. Not so uncommonly, in such papers the valuable data are still those at the core of the main experimental observation. As recently suggested by Professor Hidde Ploegh from Harvard Medical School in a recent article published in Nature^14^, *“although such extra work can provide important support for the results being presented, all too frequently it represents instead an entirely new phase of the project, or does not extend the reach of what is presented. It is often expensive and unnecessary, and slows the pace of research to a crawl.”* Unnecessary experiments are not only expensive, but also time consuming and can have a negative consequence, particularly on a young researcher’s career: “*PhD degrees are delayed, postdocs may have to wait an entire year to compete for jobs and assistant professors can miss out on promotions. […] The extra months of experiments increase costs for labs, without any obvious advantage for science*”^14^. Some of the reviewers may feel the need to ask for additional experiments, even though these experiments do not change the overall results or conclusions of the manuscript.

As previously suggested^14^, one solution is to select editors with expertise and ability to decide which of the experiments suggested by the reviewers are indeed necessary. Another useful measure would be to ask the reviewers to suggest only critical experiments addressing the validity of the manuscript’s conclusions (and to state what points of the manuscript are clarified by the requested experiments), avoiding unnecessary work that could be included in a future project. Discoveries is committed to implement these measures.**

### 7. Can we break the “glass ceiling” that excludes human materials studies from high impact journals?

Translation of mechanistic studies from tissue culture or model organisms into human subjects is perhaps one of the most daunting challenges in biomedical research. *In vivo* studies in people (i.e. clinical trials) require significant preclinical work in model organisms but the “single figure” that aims to show relevance of a model study to humans is often poorly designed and described, especially with respect to clinical parameters of tissue donors. On the other hand, many studies using materials from human subjects are relegated to relatively low impact journals and are difficult to assess by readers due to the lack of important controls, lack of subject matching and other technical weaknesses. Other studies that exclusively analyze human materials are published in modestly perceived journals due to their focus on characterization, even if such papers are destined to be widely cited by, for example, rodent studies that hunger for human relevance. Overall, the difficulties of human studies, which include recruitment challenges amidst the need for high numbers of samples to account for genetic variability, are insufficiently offset by publication in lower impact journals, where even strong human studies are generally mixed in with non-human studies of lower technical value. Sometimes human studies that provide critical foundations for years of preclinical work are published in similarly obscure journals due to lack of mechanistic or *in vivo* approaches. These limitations do not reward well-designed human studies that are absolutely critical to move fields forward, and to prevent surprises once clinical trials seem justified. High quality research on human subjects' material is absolutely essential to support top human materials research, which is a critical segue from the avalanche of models studies waiting to be proven applicable to the clinic.

Therefore, Discoveries will also consider manuscripts describing research involving human materials studies.

*Discoveries brings to the research community an outstanding editorial board that aims to address several of the innovations proposed above: there is no need to format the manuscript before submission, we have a rapid and efficient submission process, there is no need for a Cover Letter and we support the need for rules for validation of critical reagents, such as antibodies. Discoveries will aim to support high quality research on human subjects' material to provide relevance for non-human studies along with mechanistic insights into human biology and chemistry. We also aim to avoid requesting unnecessary experiments during the review process, without affecting the quality and conclusions of published manuscripts. In addition, we recognize the need of adopting the recommendations made by NCCD and other similar scientific guiding entities*.

## References

[1] Bucur Octavian, Pennarun Bodvael, Stancu Andreea Lucia, Nadler Monica, Muraru Maria Sinziana, Bertomeu Thierry, Khosravi-Far Roya (2013). Poor antibody validation is a challenge in biomedical research: a case study for detection of c-FLIP.. Apoptosis : an international journal on programmed cell death.

[2] Begley C Glenn, Ellis Lee M (2012). Drug development: Raise standards for preclinical cancer research.. Nature.

[3] Prinz Florian, Schlange Thomas, Asadullah Khusru (2011). Believe it or not: how much can we rely on published data on potential drug targets?. Nature reviews. Drug discovery.

[4] Begley C Glenn (2013). Six red flags for suspect work.. Nature.

[5] Mullard Asher (2011). Reliability of 'new drug target' claims called into question.. Nature reviews. Drug discovery.

[6] Galluzzi L, Vitale I, Abrams J M, Alnemri E S, Baehrecke E H, Blagosklonny M V, Dawson T M, Dawson V L, El-Deiry W S, Fulda S, Gottlieb E, Green D R, Hengartner M O, Kepp O, Knight R A, Kumar S, Lipton S A, Lu X, Madeo F, Malorni W, Mehlen P, Nuñez G, Peter M E, Piacentini M, Rubinsztein D C, Shi Y, Simon H-U, Vandenabeele P, White E, Yuan J, Zhivotovsky B, Melino G, Kroemer G (2012). Molecular definitions of cell death subroutines: recommendations of the Nomenclature Committee on Cell Death 2012.. Cell death and differentiation.

[7] Bucur O, Stancu A L, Khosravi-Far R, Almasan A (2012). Analysis of apoptosis methods recently used in Cancer Research and Cell Death & Disease publications.. Cell death & disease.

[8] Ploegh Hidde (2011). End the wasteful tyranny of reviewer experiments.. Nature.

[9] Kroemer G, Galluzzi L, Vandenabeele P, Abrams J, Alnemri E S, Baehrecke E H, Blagosklonny M V, El-Deiry W S, Golstein P, Green D R, Hengartner M, Knight R A, Kumar S, Lipton S A, Malorni W, Nuñez G, Peter M E, Tschopp J, Yuan J, Piacentini M, Zhivotovsky B, Melino G (2009). Classification of cell death: recommendations of the Nomenclature Committee on Cell Death 2009.. Cell death and differentiation.

[10] (2013). Frontiers in Nature: A potential opportunity to reverse the peer-review process. Neurdiness.

[11] (2008). Nature Neuroscience turns 10. Nature Neuroscience.

[12] Stephen Lagakos and the New England Journal of Medicine. New England Journal of Medicine.

[13] (2013). Independent Antibody Validation to Improve Research Quality. Science Exchange.

[14] Instructions for Authors. American Association for Cancer Research.

